# Foreign-born physicians’ perceptions of discrimination and stress in Finland: a cross-sectional questionnaire study

**DOI:** 10.1186/s12913-018-3256-x

**Published:** 2018-06-07

**Authors:** Tarja Heponiemi, Laura Hietapakka, Salla Lehtoaro, Anna-Mari Aalto

**Affiliations:** National Institute for Health and Welfare, P.O. Box 30, 00271 Helsinki, Finland

**Keywords:** Foreign-born, Physicians, Health care employees, Discrimination, Stress

## Abstract

**Background:**

Foreign-born physicians fill in the shortage of physicians in many developed countries. Labour market theory and previous studies suggest that foreign-born physicians may be a disadvantaged group with a higher likelihood of discrimination and less prestigious jobs. The present study examines foreign-born physicians’ experiences of discrimination (coming from management, colleagues and patients separately) and patient-related stress and integration-related stress, and it examines how gender, age, employment sector, country of birth, years from getting a practicing license in Finland, language problems, cross-cultural training, cross-cultural empathy, team climate and skill discretion were associated with these factors.

**Methods:**

The present study was a cross-sectional questionnaire study among 371 foreign-born physicians in Finland, aged between 26 and 65 (65% women). Analyses of covariance and logistic regression analyses were conducted to examine the associations.

**Results:**

A good team climate and high cross-cultural empathy were associated with lower likelihoods of discrimination from all sources, patient-related stress and integration-related stress. Skill discretion was associated with lower levels of integration-related stress and discrimination from management and colleagues. Language problems were associated with higher levels of integration-related stress. The biggest sources of discrimination were patients and their relatives.

**Conclusions:**

The present study showed the importance of a good team climate, cross-cultural empathy and patience, skill discretion and language skills in regard to the proper integration of foreign-born health care employees into the workplace. Good job resources, such as a good team climate and the possibility to use one’s skills, may help foreign-born employees, for instance by giving them support when needed and offering flexibility. Health care organizations should invest in continuous language training for foreign-born employees and also offer support when there are language problems. Moreover, it seems that training increasing cross-cultural empathy and patience might be beneficial.

## Background

The migration of health care professionals has been a topic of interest for many years regarding its benefits and disadvantages [1]. Foreign-born physicians fill in the shortage of physicians in many developed countries, such as the US, the UK, Australia and Canada [2]. In Finland, the number of foreign-born physicians has not traditionally been very high, but since the year 2000 the number has increased substantially [3].

According to the dual labour market theory [4], migrant employees are at high risk for low earnings, job instability, high turnover and low possibilities for promotions. Results from Germany show evidence that migrant employees face occupational segmentation, discrimination in the process of occupational attainment and a status gap compared to native employees [5]. Our previous study found evidence suggesting that foreign-born physicians could have more challenging working conditions, given that they worked more often in the primary care sector (where the work is often more stressful), they had higher levels of on-call duties and they were less likely to be in a leadership position or to have permanent employment compared to native physicians [6]. Moreover, the lack of professional support and consultation possibilities, as well as high work-related distress, burdened foreign-born physicians more than native physicians [6].

Foreign-born physicians may face more insensitivity, discrimination and isolation in their workplace [7, 8]. For example, foreign-born physicians had higher levels of threats or violence from patients compared to native physicians in Sweden [9] and foreign-born physicians have been found to face harassment from their colleagues [10]. Foreign-born physicians working in Germany have reported discrimination, difficulties in interpersonal interactions and a lack of setting-specific (language, cultural, clinical and system) knowledge [11].

One well-recognized problem among foreign-born physicians is poor language skills [12] and this has also come up in Finland [13]. A lack of a common language between patient and physician can lead to diagnostic errors and inappropriate treatment [14]. Language problems may result in communication problems and consequently predispose the physician to discrimination and integration-related problems; they may also explain (at least partly) why patients’ interaction with foreign-born physicians might be problematic.

Thus, the previous studies suggest that foreign-born physicians may be at higher risk of discrimination and having problems in interpersonal interaction, and these risks may be increased, for example, by language problems. However, there might also be factors that could attenuate these risks. For example, control over one’s job has been suggested to be especially important for foreign-born physicians [9]. Job control has been associated with lower turnover intention among foreign-born physicians [9] and it has been found to ease the potential challenges coming from working in culturally diverse teams [15]. Another concept that has been found important in culturally diverse working environments is team climate [15]. A good team climate in a work unit might leave less space for discrimination and promote good cross-cultural communication. Moreover, foreign-born physicians’ attitudes and reactions in difficult cross-cultural encounters may have an effect when communicating with colleagues and patients. For example, there are findings suggesting that cross-cultural empathy and patience, as well as cross-cultural training, might lead to less problems and confrontations [16–18].

The present study aimed to examine how gender, age, employment sector, country of birth, years from getting a practicing license in Finland, language problems, cross-cultural training, cross-cultural empathy, team climate and skill discretion were associated with foreign-born physicians’ experiences of discrimination (coming from management, colleagues and patients), patient-related stress and integration-related stress in Finland.

## Methods

### Study sample

A questionnaire survey was conducted in the autumn of 2017 in Finland. Valvira (National Supervisory Authority for Welfare and Health) delivered the data from the Terhikki central register, which contains information on Finnish health care professionals. The sample included all physicians who were born after 1944, had completed their medical education outside of Finland and who had received a license to practice medicine in Finland (*n* = 1564). For this sample we managed to get e-mail addresses (provided by the Finnish Medical Association) or postal addresses (provided by the Population Register Centre) of 1012 physicians. The main reasons for not getting some postal addresses were as follows: physicians living abroad or not having a permanent address in Finland; non-disclosure for purposes such as direct advertising, market research, opinion polls, public registers or genealogical research; or for personal safety reasons. To rule out those native Finns who had studied abroad, we excluded those whose mother tongue was one of the two official languages in Finland (Finnish or Swedish; *n* = 203) and the questionnaire was sent to 809 physicians.

An email invitation, with a link to the electronic survey, was sent to those participants whose email addresses were obtained. For those participants whose email address we did not have, a postal invitation to participate in the electronic survey was sent to their home addresses. All the participants received one reminder and on the third, and final, round an additional postal paper questionnaire (only in Finnish) was sent to those who had not responded during the first two rounds. Altogether 375 foreign-born physicians responded to the survey, the response rate being 46%. Of those, we excluded two respondents who had studied abroad and whose mother tongue was not Finnish or Swedish but who were actually born in Finland. Moreover, two respondents were excluded because they did not provide us with the information about their country of birth. Thus, the final study sample included 371 foreign-born physicians, aged between 26 and 65 (mean age: 39.1; SD = 9.09; 65% women).

The electronic questionnaire was available in Finnish, Swedish, Estonian, Russian and English languages. Ethical approval was obtained from the ethics committee of the National Institute for Health and Welfare in Finland.

### Measurements

*Discrimination* was measured by assessing whether a person had experienced personal discrimination at work during the last 12 months from 1) superiors and management, 2) colleagues and fellow employees and 3) patients/clients. The items were rated on a 5-point Likert scale ranging from 1 (*hardly ever*) to 5 (*very often or continuously*). Discrimination was defined as the unequal treatment of people (without an acceptable reason) and placing them in an unfavourable position on the basis of belonging to a certain group. These questions have previously been used among foreign-born physicians and registered nurses in Finland [13]. The statistical analyses were done separately for each source of discrimination and for the analyses the measure was dichotomized into two groups (0 = those who answered *hardly ever* and 1 = *others*).

*Patient-related stress* was measured by using three items (α = 0.82) asking participants how often the situation described in the statement had disturbed them, worried them or caused them stress during the last half-year period, rating this on a 5-point Likert scale ranging from 1 (*hardly ever*) to 5 (*very often or continuously*), higher scores indicating higher levels of stress. The items were: a) “Patients’ expectations frequently differ from those of health care personnel”, b) “Patients who complain, blame and criticize”, and c) “Patients who are unwilling to co-operate and are passive”. The pool of items included in the present study was developed from earlier studies that were carried out in the health care sector [19, 20] and previously used among foreign-born physicians [6].

*Integration-related stress* was measured by using three items (α = 0.76) asking participants how often the situation described in the statement has disturbed them, worried them or caused them stress during the last half-year period, rating this on a 5-point Likert scale ranging from 1 (*hardly ever*) to 5 (*very often or continuously*). The items were: 1) “Working in a foreign language”, 2) “Finnish workplace habits and culture” and 3) “Others do not understand my culture”.

*Language problems* were measured with seven items (α = 0.96) asking how often the respondent experienced work-related communication challenges in using Finnish language in a) face-to-face communication with patients, b) face-to-face communication with co-workers, c) workplace meetings, d) keeping up with professional literature, e) recording data in patient information systems, f) electronic communication and g) communication over the phone. The items were rated on a 5-point Likert scale ranging from 1 (*hardly ever*) to 5 (*very often or continuously*). Response alternatives also included the response option “I do not need Finnish in my work”, which was coded as *missing information*. This measure has previously been used among foreign-born physicians and registered nurses in Finland [13].

*Cross-cultural training* was assessed by asking whether the respondent had received multicultural training. Response options were a) “No”, b) “Yes, as part of my studies to become a physician”, c) “Yes, after graduating as a physician (e.g. workplace training, other further training)” and d) “Yes, I have participated in a project or development work related to multiculturalism”. For the analyses, this variable was coded as 0 = *no training* (a) and 1 = *training received* (b, c and d).

*Cross-cultural empathy* was measured by the cross-cultural emotions/empathy subscale from Bernhard’s [21] cultural competence scale (5 items; α = 0.80). Cross-cultural empathy refers to feelings and emotional reactions towards diversity, such as being multiculturally empathic and comfortable in difficult cross-cultural encounters (e.g. “In my professional interaction with patients with a migration background, I often feel unsure, angry and frustrated”; reverse coded). The items were rated on a 5-point Likert scale, ranging from 1 (*fully disagree*) to 5 (*fully agree*), higher values indicating higher empathy and patience.

*Team climate* was measured with four items (α = 0.90) from the Team Climate Inventory (TCI) [22, 23]. Subscale participative safety was used, which relates to active involvement in group interactions wherein the predominant interpersonal atmosphere is one of non-threatening trust and support. The items were rated on a 5-point Likert scale, ranging from 1 (*fully disagree*) to 5 (*fully agree*), higher scores indicating a better team climate.

*Skill discretion* was measured by using the skill discretion scale (3 items; α = 0.75) derived from Karasek’s Job Content Questionnaire (JCQ) [24]. The scale measures the flexibility permitted to workers in deciding what skills to employ (e.g. “My job requires that I learn new things”). The items were rated on a 5-point Likert scale, ranging from 1 (*fully disagree*) to 5 (*fully agree*).

*Employment sector* was categorized into three groups: the municipal sector (e.g. primary care), the state sector (e.g. hospitals) and the private sector. *Country of origin* was asked in the questionnaire with an open-ended question and categorized as those coming from a) Estonia, b) the Russian Federation / the former Soviet Union, c) other EU/EEA countries and d) other countries.

### Statistical analysis

The effects of independent variables on patient-related stress and integration-related stress were examined with the analyses of covariance (in separate analyses). The analyses were conducted in one step, including the effects of gender, age, employment sector, country of birth, years from getting a practicing license, language problems, multicultural training, cross-cultural empathy, team climate and skill discretion in the model. For the dichotomous discrimination variables (separate for each source), logistic regressions were applied with the same model as mentioned above. All analyses were performed using SPSS software, version 24.0.

## Results

### A description of the sample

The characteristics of the study population can be seen in Table 1. There were more women respondents than men respondents and most worked for a municipal employer. The majority of respondents came from Estonia (38%) or the Russian Federation / the former Soviet Union (30%). Discrimination from patients and their relatives was the most common source of discrimination, whereas discrimination coming from superiors and management was the least often experienced source of discrimination. The mean time elapsed since getting a license to practice a profession in Finland was five years.

**Table 1:** The characteristics of the study sample

	n	%
Gender		
Women	240	65.2
Men	128	34.8
Sector		
Municipal (primary care)	280	76.5
State (hospitals)	34	9.3
Private	52	14.2
Country of birth		
Estonia	142	38.3
Russian Federation / the former Soviet Union	111	29.8
Other EU/EEA country	64	17.3
Other country	54	14.6
Discrimination from superiors		
Hardly ever	302	82.5
Not often	26	7.1
Sometimes	24	6.6
Fairly often	11	3.0
Very often or continuously	3	0.8
Discrimination from colleagues		
Hardly ever	263	72.1
Not often	58	15.9
Sometimes	30	8.2
Fairly often	10	2.7
Very often or continuously	4	1.1
Discrimination from patients		
Hardly ever	161	44.1
Not often	104	28.5
Sometimes	79	21.7
Fairly often	18	4.9
Very often or continuously	3	0.8
Cross-cultural training		
No	310	84.2
Yes, as part of my studies to become a physician	21	5.7
Yes, after graduating as a physician	31	8.5
Yes, as part of development work or a project	6	1.6
	Mean	SD
Age	39.1	9.13
Years from getting a practicing license in Finland	5.2	3.11
Patient-related stress^a^	2.41	0.81
Integration-related stress^a^	1.67	0.73
Language problems^a^	2.03	1.20
Cross-cultural empathy^a^	3.75	0.88
Team climate^a^	4.16	0.83
Skill discretion^a^	4.60	0.53

^a^The scale ranged between 1 and 5

Most respondents (84%) had not received any kind of cross-cultural training; the most common type of cross-cultural training occurred after graduating as a physician. Mean stress levels were quite low among foreign-born physicians; their patient-related stress levels were higher than integration-related stress levels. The respondents rated their language problems quite low. Job resources were rated rather high; skill discretion levels were a bit higher than team climate levels. Cross-cultural empathy was also rated a bit over the average.

### Factors associated with patient-related stress

The results of the analyses of covariance for patient-related stress showed that gender, age, cross-cultural empathy and team climate were significantly associated with patient-related stress (Table 2). Women and younger respondents had higher levels of patient-related stress compared to their counterparts. Higher cross-cultural emotion levels and a better team climate were associated with lower levels of patient-related stress.

**Table 2:** The results of the analyses of covariance for patient-related stress and integration-related stress

	F	p
Patient-related stress		
Gender	5.26	0.022
Age	16.33	< 0.001
Employment sector	0.69	0.503
Country of birth	1.42	0.238
Years from practicing licence	0.03	0.866
Language problems	2.76	0.098
Cross-cultural training	1.61	0.206
Cross-cultural empathy	16.80	< 0.001
Team climate	3.96	0.048
Skill discretion	0.19	0.663
R^2^	0.12	
Integration-related stress		
Gender	0.52	0.471
Age	7.55	0.006
Employment sector	1.35	0.261
Country of birth	4.89	0.002
Years from getting a practicing licence	0.35	0.555
Language problems	19.74	< 0.001
Cross-cultural training	0.56	0.457
Cross-cultural empathy	6.91	0.009
Team climate	31.61	< 0.001
Skill discretion	4.55	0.034
R^2^	0.26	

### Factors associated with integration-related stress

Analyses showed that age, country of birth, language problems, cross-cultural empathy, team climate and skill discretion were significantly associated with integration-related stress (Table 2). Older respondents had higher levels of integration-related stress. Those coming from Estonia or the Russian Federation / the former Soviet Union had lower levels of integration-related stress, whereas those coming from other EU/EEA countries or other countries had higher levels of integration-related stress. Language problems were associated with higher levels of integration-related stress, whereas higher levels of cross-cultural empathy, better team climate and higher skill discretion were associated with lower levels of integration-related stress.

### Factors associated with discrimination

Logistic regression analyses showed that respondents coming from Russia, other EU/EEA countries and other countries were more likely to experience discrimination from all sources than respondents coming from Estonia (Table 3). Moreover, a better team climate was associated with the lower likelihood of discrimination from all sources. The longer time from getting a practicing licence from Finland was associated with the higher likelihood of discrimination from management and patients. Those who had had cross-cultural training were more likely to experience discrimination from management. Higher levels of cross-cultural empathy were associated with the lower likelihood of discrimination from management. Higher levels of skill discretion were associated with lower likelihood of discrimination from management and colleagues.

**Table 3:** The results of the logistic regression analysis for discrimination from different sources (presenting odds ratios [ORs] and their 95% confidence intervals [95% CIs])

	Discrimination from management^a^			Discrimination from colleagues^a^			Discrimination from patients^a^		
	OR	(95% CI)	p	OR	(95% CI)	p	OR	(95% CI)	p
Gender			0.064			0.323			0.198
Men	1			1			1		
Women	1.99	0.96–4.10		1.33	0.76–2.34		0.72	0.44–1.19	
Age	0.96	0.67–1.38	0.836	0.90	0.67–1.21	0.505	0.79	0.61–1.03	0.077
Employment sector			0.352			0.433			0.675
Municipal (primary care)	1			1			1		
State (hospitals)	0.89	0.30–2.65		1.62	0.68–3.90		0.71	0.32–1.57	
Private	0.45	0.15–1.33		1.41	0.65–3.06		1.06	0.54–2.11	
Country of birth			0.025			0.003			< 0.001
Estonia	1			1			1		
Russia^b^	1.85	0.77-4.46		2.32	1.13–4.73		3.53	1.94–6.43	
Other EU/EEA country	3.00	1.20–7.46		3.57	1.66–7.69		3.12	1.56–6.26	
Other country	4.59	1.58–13.34		3.68	1.55–8.74		4.58	2.05–10.23	
Years from getting a practicing licence	1.59	1.16–2.17	0.004	1.11	0.84–1.47	0.467	1.32	1.01–1.73	0.044
Language problems			0.698			0.153			0.462
No	1								
Yes	1.07	0.76–1.50		1.21	0.93–1.58		1.10	0.86–1.40	
Cross-cultural training			0.031			0.086			0.941
No	1			1			1		
Yes	2.61	1.09–6.24		1.88	0.92–3.86		1.02	0.53–1.96	
Cross-cultural empathy	0.71	0.52–0.97	0.032	1.02	0.78–1.33	0.889	1.04	0.82–1.30	0.764
Team climate	0.44	0.31–0.61	< 0.001	0.57	0.43–0.76	< 0.001	0.69	0.53–0.91	0.008
Skill discretion	0.67	0.48–0.91	0.012	0.63	0.47–0.85	0.002	0.88	0.67–1.17	0.378
R^2^	0.31			0.24			0.16		

Continuous variables were used as continuous standardized variables and the model ORs presented indicate the likelihood of passing from *no discrimination* to *discrimination*, compared to one standard deviation change in continuous independent variables

*OR* odds ratio, *CI* confidence interval

^a^Discrimination variables were dichotomized (as 0 = *hardly ever* and 1 = *others*) for these analyses

^b^Russian Federation / the former Soviet Union

## Discussion

The present study examined experiences of discrimination and stress among foreign-born physicians in Finland. We found that work-related resources were of importance for foreign-born physicians: good team climate was associated with lower levels of patient-related stress, integration-related stress and discrimination from management, colleagues and patients. Also, high levels of skill discretion were associated with the lower likelihood of integration-related stress and discrimination from management and colleagues. Moreover, cross-cultural empathy was associated with lower levels of patient-related stress, integration-related stress and discrimination from management. Language problems were associated with higher levels of integration-related stress. The biggest sources of discrimination were patients and their relatives. Older respondents experienced more integration-related stress than younger respondents.

The present study highlights the importance of a good team climate in cross-cultural workplaces as this was associated with all of our outcomes. However, a previous study showed that native Finnish physicians working in culturally diverse work units are at higher risk of experiencing a poor team climate compared to physicians working with predominantly native colleagues [15]. Thus, cross-cultural workplaces need to emphasize and actively promote a good team climate, given that it is essential but easily challenged in cross-cultural encounters. A good team climate has also been previously associated with delivering high quality care, for example, through professionals sharing support, commitment and objectives [25, 26]. When the team climate is good, professionals can freely communicate with each other about their tasks and expertise; they share information and everybody feels accepted and understood [22, 23]. According to our results it seems that this is crucial in order for foreign-born professionals to be able to integrate and succeed in Finnish working life. A good team climate and possibilities to use one’s skills may help foreign-born employees, for instance by giving support when needed and offering flexibility. Maintaining a good team climate in cross-cultural working teams may be a challenge and may require constant effort, but it may also bring great benefits for employees and organizations.

Also, another job resource, namely skill discretion, seemed important for foreign-born physicians. This is congruent with the previous findings wherein job resources were closely related to skill discretion, showing, for example, that control over work pace is associated with decreased levels of turnover intentions among foreign-born physicians [9]. Possibilities to control one’s job have also been found to mitigate the potential challenges coming from working in culturally diverse work teams [15]. A previous study has shown that healthy conditions for employees allow employees to spend as much energy as possible on learning and acquiring new skills [27]. Job redesigns, aiming at increasing job variety, exchange programmes across hospitals and wards, and mentoring programmes have all been suggested as interventions to promote new skills and to provide new work experiences [27].

According to our results, cross-cultural emotional empathy among foreign-born physicians may help them when encountering patients from different cultures and help them to integrate into a foreign country. Cultural empathy has previously been related to a problem-solving strategy that is associated with remaining calm in stressful situations [18] and involving the trait of emotional intelligence. Thus, it is logical that emotional empathy and patience may also be important for foreign-born physicians when facing problems with patients and in difficult interpersonal relationships. Therefore, it seems that training increasing cross-cultural empathy and patience might be beneficial. Cross-cultural empathy and patience can be taught; for example, even a short one-hour formal lecture has been found to induce positive change in cultural empathy [16].

In our study, patients and their relatives were the most common source of discrimination; 27% of the participating physicians described having experienced it *sometimes* or *often*, whereas the corresponding numbers for discrimination from superiors and management was 10% and from colleagues and co-workers it was 12%. Also, previous studies have shown that foreign-born physicians face discrimination, insensitivity, racism and rejection from patients and colleagues [e.g. 7, 11]. Threats and violence from patients have been found to be more common among foreign-born physicians than among native physicians [9].

Our results suggest that older foreign-born physicians struggle more with working in a foreign culture and in a foreign language than their younger counterparts. This may be related to our finding that those whose licence to practice in Finland was granted a longer time ago were more likely to experience discrimination from management and patients compared to those who had had received their licence more recently. Thus, it seems that older foreign-born professionals especially need extra support in the workplace.

According to our results, language skills are important in helping the foreign-born physicians’ integrate into working life. Previous studies have emphasized the relevance of language skills for foreign-born physicians [12, 13, 28] and the negative ramifications of poor language skills for patient care [14, 29, 30]. Therefore, it is important that health care organizations provide language training and support for their foreign-born employees. In addition, public authorities and employment offices should put much more effort into language training for migrants in order to enable the proper integration to society and working life.

In interpreting the present results, it is important to note some limitations. The present study relied on self-reported measures, which may lead to problems associated with an inflation of the strengths of relationships and with the common method variance. To minimize problems with self-reports, we used measures that showed good reliability. However, self-rated measures may be biased, for example, patient-related stress may be influenced by respondents’ experiences of discrimination. In addition, although we controlled for age, gender, employment sector, country of birth and years from getting a practicing licence in Finland, we cannot rule out the possibility of residual confounding. Because the present study was cross-sectional, we cannot draw any causal inferences. Moreover, the sample is not totally representative of foreign-born physicians practicing in Finland, because it was not possible for us to get information about citizenship or country of birth from the registers when drawing the sample. Therefore, we used having a mother tongue other than Finnish or Swedish as a criterion and excluded physicians whose mother tongue was Finnish or Swedish (official languages in Finland). This was done to ensure that our sample would not include those native Finnish physicians who had studied abroad. Consequently, our sample does not include foreign-born physicians born in Sweden; however, they constitute only a small portion of foreign-born physicians in Finland (in 2010 they accounted for approximately 2% of foreign-born physicians working in Finland [31]).

## Conclusions

The present study showed the importance of good team climate, cross-cultural empathy and patience, skill discretion and language skills for the proper integration of foreign-born health care employees into the workplace. Maintaining a good team climate in cross-cultural working teams and giving foreign-born employees possibilities to use their skills may be a challenge and may require constant effort, but it may also bring great benefits for employees and organizations. Health care organizations should also invest in offering training for their foreign-born employees, for example, training related to language, and emotional patience and empathy. Future studies should examine the best ways for health care organizations to implement training and interventions in order to increase foreign-born employees’ integration into the workplace.

## Abbreviations

JCQ: Job Content Questionnaire
TCI: Team Climate Inventory
